# Emergency Medical Technicians' Experiences of the Challenges of Prehospital Care Delivery During the COVID-19 Pandemic: A Qualitative Study

**DOI:** 10.4314/ejhs.v31i6.6

**Published:** 2021-11

**Authors:** Mohammad Parvaresh-Masoud, Masoomeh Imanipour, Mohammad Ali Cheraghi

**Affiliations:** 1 Department of Critical Care and Management Nursing, School of Nursing and Midwifery, Tehran University of Medical Sciences, Tehran, Iran; 2 Nursing and Midwifery Care Research Center, Tehran University of Medical Sciences, Tehran, Iran

**Keywords:** COVID-19, SARS-CoV-2, Delivery of Health Care, Emergency Medical Services, Emergency Medical Technicians

## Abstract

**Background:**

Exploring emergency medical technicians' (EMTs) experiences of COVID-19 epidemic, help to identify the challenges they face in their daily work and develop strategies that address these challenges. This study aimed to explore EMTs' experiences of the challenges of prehospital care delivery during the COVID-19 pandemic.

**Methods:**

This qualitative study was conducted in March-July 2020 using conventional content analysis approach. Fifteen EMTs were purposively selected from the Emergency Medical Services (EMS) Center in Qom, Iran. For data collection, semi-structured interviews were conducted until data saturation was reached.

**Results:**

EMTs' experiences of the challenges of prehospital care delivery during the COVID-19 pandemic were classified into three main categories including “restless society”, “difficult care delivery conditions”, and “unprepared organization”. The emergent subcategories were “need for information”, “limited perception of the COVID-19 risk”, “obsessive use of disinfectants”, “fear over the transmission of COVID-19 to self and others”, “burnout due to heavy workload”, “altered communication with hospital staff”, “ethical conflicts”, “lack of a definite treatment plan”, “lack of protective equipment”, “staff shortage due to the affliction of EMTs by COVID-19”, and “inadequate support by authorities”.

**Conclusion:**

During COVID-19 pandemics, EMTs face many challenges including emotional and occupational stress, social strains, risk of affliction by infections, heavy workload, and ethical conflicts and hence, experience difficulties in quality care delivery. Developing appropriate strategies, guidelines, and policies are needed to effectively manage these challenges and improve the quality of prehospital care delivery in COVID-19 epidemic.

## Introduction

Coronavirus disease 2019 (COVID-19) pandemic, as an unprecedented public health crisis, is producing a huge health care burden with millions of cases and thousands of deaths (1). In response to the pandemic, several new strategies and policies have been used by government, including social distancing, restriction of unnecessary travels, and suspension of the activities of educational settings (2). These strategies had positive effects on disease management, but caused serious problems and challenges, particularly for poor and vulnerable people (3). COVID-19 has also negatively affected healthcare professionals and put them at risk for different problems (4). Healthcare professionals currently have a heavy workload and are at risk for severe COVID-19 and subsequent death (5). Most of them are concerned about protecting themselves and their families against COVID-19 (6). They experience considerable stress and challenges due to the risk of developing COVID-19, witnessing the death of afflicted patients, having inadequate protection against the disease, having limited communication with their families, and experiencing prejudice and frustration (7). Moreover, their capabilities cannot fully be used for long period of time (8).

COVID-19 also significantly affected the delivery of prehospital emergency medical services (EMS). Like other healthcare professionals, emergency medical technicians (EMTs) have critical roles in the management of epidemics and disasters. However, epidemics put them at high risk of infection, physical and mental injuries, and death (9). During epidemics, public concerns and fears significantly increase the demand for healthcare services and hence, the number of contacts with EMS dispatch centers significantly increases, resulting in significant increase in EMTs' workload (10–11). Moreover, EMTs should work in the difficult, unsafe, and uncontrollable conditions of prehospital settings, including homes, public places, and criminal and accident scenes (12). Unlike care delivery in controllable environments such as hospital settings, care delivery in prehospital settings is associated with many different problems and challenges such as the overcrowding of the environment, need for rapid decision making, need for taking emergency measures without having adequate information, and problems associated with patient transfer (13). Moreover, EMTs experience moral distress due to the ethical conflicts related to taking informed consent, protecting patient privacy, giving bad news, and managing patients' refusal of treatments or transfer (14).

Previous studies reported that in epidemics, healthcare providers experience high levels of occupational strain and face different challenges due to the high risk of infection, inadequate personal protective equipment, heavy workload, staff shortage, uncertainties, prejudice, patients' negative emotional reactions, separation from families, and job burnout. COVID-19 pandemic has also added the challenges related to the triage and allocation of limited resources and medical decision making in critical conditions (15–16). These problems and challenges can cause EMTs physical and mental health problems such as stress, anxiety, depression, sleeplessness, denial, anger, and fear. Mental health problems in turn not only affect their decision-making abilities, but also may cause them permanent physical and mental health problems even after the end of epidemics (6–7, 9, 11, 17–18). These problems and challenges can affect EMTs' ability to provide quality care to patients with suspected COVID-19 (11). The effective management of these problems and challenges in the COVI-19 pandemic depends on their deep understanding. However, there are limited data about these challenges in COVID-19 pandemic. The present study was conducted to explore EMTs' experiences of the challenges of prehospital care delivery during the COVID-19 pandemic.

## Methods

This qualitative content analysis study was conducted in March-July 2020. Study setting was the EMS Center in Qom, Iran. Participants were fifteen EMTs with the experience of care delivery to patients with COVID-19 since the diagnosis of the first case in Iran in February 19, 2020. Sampling was done purposively. Selection criteria were the experience of care delivery to patients with COVID-19 and agreement for participation.

After approval of the study by the ethics committee of Tehran University of Medical Sciences, Tehran, Iran, data were collected via semi-structured interviews held by the first author in participants' preferred place. Interviews were started using broad open-ended questions about the experience of care delivery in epidemics and the challenges of care delivery to patients with COVID-19. The length of the interviews varied from 35 to 75 minutes. Data collection was continued up to data saturation, i.e. when the data became repetitive and no new data were obtained from the interviews. Although data saturation was achieved with twelve interviews, three more interviews were held to ensure saturation.

Concurrently with data collection, the data were analyzed using Graneheim and Lundman's five-step conventional content analysis approach (19). Immediately after each interview, it was transcribed word by word and the transcript was reviewed to grasp its main ideas. Then, each interview transcript was considered as the unit of analysis and meaning units were identified and coded. According to their similarities, generated codes were grouped into subcategories. Finally, subcategories were compared and grouped to develop larger categories and identify the latent content of the data.

Trustworthiness was established using Guba and Lincoln's criteria (20). To ensure credibility, the first author had prolonged engagement with the data and participants, attended participants' workplace, and attempted to improve his professional knowledge and competence for doing qualitative studies through studying qualitative research textbooks, seeking advice from experienced qualitative researchers, and closely adhering to the steps of doing qualitative research. Dependability was ensured through member checking, in which generated codes of some interviews were provided to the intended participants to ensure the accuracy of the codes. Codes which were not congruent with participants' experiences were revised. Moreover, excerpts from the data and their corresponding codes and categories were provided to several experts in EMS and qualitative research who confirmed the accuracy of data analysis. To ensure confirmability, detailed data were provided about participants' age, gender, work experience, and educational level as well as the characteristics of the study setting. These data help the readers of the article better understand the context of the study. Transferability was also ensured through sampling with maximum variation in terms of participants' age, work experience, educational level, and workplace. The time and the place of interviews were arranged according to participants' preferences.

**Ethical consideration:** Ethical approval of the study was obtained from ethics committee of Tehran University of Medical Sciences, Tehran, Iran (decree number: IR.TUMS.VCR.REC.1397.642). At the beginning of the interviews, participants were informed about the study aim, interview process, confidential data management, and voluntariness of participation. Participation in the present study was completely voluntary and informed consent was obtained from all participants.

## Results

All participants were male and were working in the EMS dispatch center or urban or road EMS stations (Table 1). During data analysis eleven subcategories and three main categories, i.e., restless society, difficult care delivery conditions, and unprepared organization were emerged (Table 2).

**Table 1:** Participants' characteristics

No	Educational level	Age (Years)	Work experience in prehospital EMS (Years)	Workplace	History of COVID-19 affliction
1	Diploma	51	27	Road EMS station	No
2	Associate degree in Anesthesia	38	16	Dispatch center	No
3	Associate degree in medical emergency	25	2	Road EMS station	No
4	Associate degree in medical emergency	32	8	Dispatch center	No
5	Associate degree in medical emergency	35	10	Urban EMS station	Yes
6	Bachelor's degree in medical emergency	26	2	Road EMS station	No
7	Bachelor's degree in medical emergency	26	2	Urban EMS station	No
8	Bachelor's degree in medical emergency	27	3	Road EMS station	Yes
9	Bachelor's degree in medical emergency	29	5	Urban EMS station	No
10	Bachelor's degree in medical emergency	30	5	Road EMS station	No
11	Bachelor's degree in nursing	35	7	Road EMS station	Yes
12	Bachelor's degree in nursing	37	12	Urban EMS station	No
13	Bachelor's degree in nursing	38	18	Urban EMS station	No
14	Master's degree in nursing	36	10	Road EMS station	No
15	Master's degree in nursing	44	18	Urban EMS station	Yes

**Table 2:** EMTs' experiences of the challenges of prehospital care delivery during the COVID-19 pandemic

Subcategories	Categories
Need for information	**Restless society**
Limited perception of the COVID-19 risk	
Obsessive use of disinfectants	

Fear over the transmission of COVID-19 to self and others	**Difficult care delivery conditions**
Burnout due to heavy workload	

Altered communication with hospital staff	
Ethical conflicts	

Lack of a definite treatment plan	**Unprepared organization**
Lack of protective equipment	
Staff shortage due to the affliction of EMTs by COVID-19	
Inadequate support by authorities	

**Restless society:** The COVID-19 pandemic caused significant changes in people's behaviors. People had limited knowledge about the disease and hence, showed different and conflicting behaviors towards it. They desperately needed information about COVID-19 while they were exposed to a wide range of accurate and inaccurate information in the social media. The desperate need for information and the unavailability of accurate information caused people to contact EMS even in case of minor symptoms. All these problems had caused a state of restlessness in the society which was associated with challenges for EMTs. The three subcategories of this category were need for information, limited perception of the COVID-19 risk, and obsessive use of disinfectants.

**Need for information:** COVID-19 affected the different aspects of people's lives and caused them different concerns. Therefore, they sought information about the disease and its effects on their physical and mental health. These conditions made people seek for a reliable source information. The most accessible source of information was EMS. Therefore, the number of contacts with EMS and the number of EMS missions considerably increased.

*“Most people who contacted EMS needed assessment and reassurance. People contacted EMS even in case of minor symptoms to know whether they have COVID-19” (P. 9).*

**Limited perception of the COVID-19 risk:** People's behaviors towards COVID-19 widely varied mostly due to the conflicting policies and guidelines which were frequently revised. These problems reduced people's perceived risk of COVID-19 and resulted in their inattention and non-adherence to guidelines which in turn increased the prevalence of COVID-19 and the number of EMS contacts, particularly in the early days of the pandemic.

*“One day, we were dispatched on a mission to a home with a patient suspected of having COVID-19. We asked other family members to take special care and informed them about the high risk of transmission. One week after, we were dispatched on a mission to the same home in order to transfer another patient with COVID-19” (P. 5).*

**Obsessive use of disinfectants:** During the COVID-19 pandemic, people resorted to disinfectants and widely used them in order to reduce the risk of transmission. The wide and inappropriate use of disinfectants caused serious health problems such as skin lesions, eye problems, and respiratory disorders which resulted in frequent contacts with EMS and significant challenges for EMTs.

*“We were dispatched on a mission to a woman who had combined hydrochloric acid and sodium hypochlorite for cleaning the toilet. The inhalation of the resultant gases had caused her severe dyspnea and coughs” (P. 12).*

**Difficult care delivery conditions:** Participants reported that they experienced considerable physical and mental strains due to the high risk of affliction by COVID-19, limited personal protective equipment, heavy workload, staff shortage, separation from family, and alteration in their communications with hospital staff. The four subcategories of this category were fear over the transmission of COVID-19 to self and others, burnout due to heavy workload, altered communication with hospital staff, and ethical conflicts.

**Fear over the transmission of COVID-19 to self and others:** The contagiousness of COVID-19 and inadequacy of information about it caused EMTs great fear and concern. Despite using protective clothes and equipment, they had fear over COVID-19 transmission to themselves, their colleagues, and their families. EMTs who lived with their families were also highly concerned over transmitting COVID-19 to their family members, particularly their children and parents.

*“I experience anxiety after each shift due to the possibility of taking the virus with myself to home. I have a little baby and have fear over his affliction by COVID-19” (P. 14).*

**Burnout due to heavy workload:** In the first days of the pandemic, workload was very heavy, many contacts were made with EMS, and the number of missions was high. The necessity to use protective clothes, deliver care to criticallyill patients, and work overtime due to colleagues' affliction by COVID-19 also increased their workload.

*“The number of contacts and missions had increased considerably. Our EMS station covers many villages. During our 48-hour shift, we were dispatched on 7–8 missions and hence, I had to drive ambulance for long hours. One time, we fell asleep due to severe fatigue and did not notice the call for a mission” (P. 3).*

**Altered communication with hospital staff:** Most participants highlighted the importance of effective communication between EMTs and hospital staff and noted that their current communications were not effective, particularly in stressful conditions. There were limited hospitals for care delivery to patients with COVID-19, particularly at the first weeks of the pandemic and hence, EMTs had problems in gaining admission to hospitals for patients with and without COVID-19, resulting in conflicts between EMTs and hospital staff.

*“After experiencing considerable stress during patient care delivery and patient transfer, we had to gain admission to hospital for the intended patient. I remember that we transferred a patient with suspected COVID-19 to a hospital, where they easily said that they didn't admit that patient and said that we had to take him to another hospital” (P. 9).*

**Ethical conflicts:** Prehospital EMS delivery is a complex stressful job. The COVID-19 pandemic added to that stress and caused EMTs ethical conflicts in decision making about accurate and quick care delivery to patients. Alongside with self-protection, EMTs needed to protect patients' rights and protect them against injuries. Prioritizing self or patient caused EMTs ethical conflicts.

*“One time, we were dispatched on a mission to a patient with severe dyspnea who could hardly breathe. I knew that I had to perform airway management interventions; but I had fear over contamination with the virus. I didn't know what I had to do in such conditions” (P. 15).*

**Unprepared organization:** The EMS system needs to develop and use strategies and plans for increasing its care delivery capacity in critical and emergency conditions. Participants noted that adequate number of qualified staff, adequate personal protective equipment for them, and clear evidence-based guidelines can increase the effectiveness of care services. The four subcategories of this category were lack of a definite treatment plan, lack of protective equipment, staff shortage due to the affliction of EMTs by COVID-19, and inadequate support by authorities.

**Lack of a definite treatment plan:** Uncertainty over treatment and care services, especially in the early days of the COVID-19 pandemic, was challenging for EMTs and caused them despair.

*“When we were dispatched on missions to patients with suspected COVID-19, I didn't really know what I had to do. There was neither a clear guideline nor previous experience” (P. 7).*

**Lack of protective equipment:** Due to the sudden onset of the COVID-19 pandemic, there was limited protective equipment for EMTs and other healthcare providers in the early days of the pandemic.

*“They gave me an N95 mask and a protective cloth for a whole shift. What I had to do if I was dispatched on two COVID-19 missions?” (P. 12).*

**Staff shortage due to the affliction of EMTs by COVID-19:** The COVID-19 pandemic significantly increased the need for qualified healthcare providers. At the same time, the number of available EMTs for care delivery significantly decreased due to factors such as their affliction by the disease, fear over affliction, family members' request for not working in critical conditions, job abandonment due to the lack of protective equipment, and staying home for care delivery to afflicted family members.

*“In our EMS station, two colleagues were simultaneously afflicted by COVID-19 and we had to fill in for them. We couldn't ask help from other stations because almost all stations had the same problem” (P. 5).*

**Inadequate support by authorities:** Most participants were dissatisfied with the support they received from managers and authorities. They expected managers and authorities to provide them with the necessary facilities and services in order to enable them to provide quality care in the stressful conditions of the COVID-19 pandemic.

*“Is it possible that they issue a guideline one day and another guideline the day after? What is the advantage of such guidelines except for confusing the technicians?” (P. 13).*

## Discussion

This study revealed that EMTs faced many different challenges during care delivery to patients with suspected COVID-19, during patient transfer to hospital, and after their missions. Our findings showed a considerable increase in the number of contacts with EMS centers during the COVID-19 pandemic. Uncertainties during epidemics have great potential for causing fear and anxiety. Consequently, the number of contacts with EMS significantly increases which in turn increases EMTs' workload (10, 18). Increased number of contacts with EMS centers is associated with an increase in the number of EMS missions which is a major challenge for EMTs. The same conditions were observed during swine flu pandemic in 2009 (21).

The psychological strain of COVID-19 can also negatively affect individuals, families, and societies and can lead to the inappropriate use of healthcare resources and services. Different studies reported lack of knowledge as a main factor contributing to public fear of COVID-19, particularly in the early days of the pandemic (22, 23). One of the first outcomes of public fear is increased number of contacts with EMS and increased number of EMS missions (10).

Present study also showed that people's limited perception of the COVID-19 risk was a care delivery challenge for EMTs. The experiences of the SARS, flu, and swine flu epidemics show that the effectiveness of epidemic management strategies largely depends on people's perceived risk of the epidemic (24). The results of a former study in Iran showed that culture and religion significantly affect risk perception among Iranians (25). These findings highlight the necessity of providing quality and timely culturally appropriate educations in epidemics in order to create a healthy level of emotional fear in society and thereby, promote people's adherence to educations and protocols.

Healthcare providers experience different stressors during epidemics. Factors such as the increasing prevalence of the disease, its prolonged course, limited personal protective equipment, fear over transmitting the disease to the family, colleagues' affliction by the disease, and the necessity to make difficult decisions about the allocation of limited resources to different patients impose heavy psychological strain on healthcare providers (26). EMTs are in the frontline of care delivery to patients with COVID-19 and hence, are at high risk of affliction by the disease. Therefore, they experience deep fear and anxiety over affliction by the disease and its transmission to their families. Previous studies also reported the same finding (27, 28). Our findings also showed extreme fatigue and heavy workload among the main challenges of care delivery for EMTs during the COVID-19 pandemic. The sudden increase in the request for healthcare services causes tensions in healthcare systems and imposes heavy workload on healthcare providers (29). It has been previously showed a significant increase in the workload of EMTs during the H1N1 pandemic (30). Generally, public health emergencies, such as flu epidemics, impose heavy workload on healthcare providers (31) and may lead to their job abandonment.

In this study, participants noted that the number of hospitalization beds for patients with COVID-19 was very low and hence, they experienced problems such as lengthy missions, fatigue, conflicts with hospital staff, and staff and patient dissatisfaction. Effective planning for quality care delivery during all epidemics is among the key responsibilities of healthcare systems. All hospitals should be ready to admit and provide services to patients with and without COVID-19 (32). However, previous studies showed challenges in policy making regarding the effective management of epidemics (27, 30, 32). We also found ethical conflicts as a challenge of care delivery in the COVID-19 pandemic. EMTs work in complex, stressful, and unsafe conditions. They need to work in emergency conditions and hence, experience high levels of physical and emotional stress associated with stabilizing patients' conditions, reducing their anxiety, and ensuring care continuity (33–34). A study showed that public health emergencies and care delivery to critically-ill patients with COVID-19 or other respiratory disorders can alter routine ethical processes (32). Lack of personal protective equipment was a main challenge of care delivery for EMTs in the present study and caused them fear, concern, and lack of motivation for work. Previous studies also reported lack of personal protective equipment during epidemics and disasters (35–37). Inadequate personal protective equipment can put healthcare providers at risk for health problems and thereby, negatively affect the overall performance of the healthcare system (36).

EMTs have critical roles in care delivery during epidemics. However, they may be inaccessible or have limited motivation for work during epidemics due to factors such as health problems, family commitments, and fear over contamination (38–39). A study showed that the two important predictors of EMTs' willingness to work were a sense of safety at work during an epidemic and receiving adequate protective services for themselves and their family members (36). Recall bias from study participants during the data collection and lack of generalizability of the findings to other EMTs in different geo-cultural contexts, due to small sample size and participants' characteristics are the limitations of this study.

In conclusion, it seems that EMTs face different challenges during prehospital care delivery in the COVID-19 pandemic. These challenges are related to people and clients, EMTs and other healthcare providers, and healthcare organizations, and significantly affect the quality of prehospital care delivery by EMS. Healthcare managers, authorities, and policy makers need to pay careful attention to these challenges and develop effective strategies for their management in order to minimize their negative effects on EMTs and improve the quality of their care services.
